# Long Non-Coding RNAs as Molecular Biomarkers in Cholangiocarcinoma

**DOI:** 10.3389/fcell.2022.890605

**Published:** 2022-04-27

**Authors:** Yanhua Wu, Khizar Hayat, Yufei Hu, Jianfeng Yang

**Affiliations:** ^1^ Department of Gastroenterology, The Fourth School of Clinical Medicine, Zhejiang Chinese Medical University, Hangzhou, China; ^2^ Department of Gastroenterology, International Education College of Zhejiang Chinese Medical University, Hangzhou, China; ^3^ Department of Gastroenterology, Affiliated Hangzhou First People’s Hospital, Zhejiang University School of Medicine, Hangzhou, China; ^4^ Key Laboratory of Integrated Traditional Chinese and Western Medicine for Biliary and Pancreatic Diseases of Zhejiang Province, Hangzhou, China

**Keywords:** cholangiocarcinoma, lncRNA, biomarker, diagnosis, prognosis, therapeutic target

## Abstract

Cholangiocarcinoma (CCA) is a biliary system cancer that has the characteristics of strong invasiveness, poor prognosis, and few therapy choices. Furthermore, the absence of precise biomarkers for early identification and prognosis makes it hard to intervene in the early phase of initial diagnosis or recurring cholangiocarcinoma following surgery. Encouragingly, previous studies found that long non-coding RNA (lncRNA), a subgroup of RNA that is more than 200 nucleotides long, can affect cell proliferation, migration, apoptosis, and even drug resistance by altering numerous signaling pathways, thus reaching pro-cancer or anti-cancer outcomes. This review will take a retrospective view of the recent investigations on the work of lncRNAs in cholangiocarcinoma progression and the potential of lncRNAs serving as promising clinical biomarkers and therapeutic targets for CCA.

## Introduction

Cholangiocarcinoma (CCA) is an extremely aggressive malignancy of the bile duct epithelium. Depending on the anatomical site of origin, CCA can be stratified into three categories: intrahepatic cholangiocarcinoma (iCCA), perihilar cholangiocarcinoma (pCCA), as well as distal cholangiocarcinoma (dCCA) (Rizvi and Gores, 2013). Cholangiocarcinoma is the 2nd most prevalent primary hepatobiliary cancer after HCC (hepatocellular carcinoma). It accounts for about 3% of all gastrointestinal malignant tumors (Banales et al., 2016). Cholangiocarcinoma, particularly intrahepatic cholangiocarcinoma, has become more common in the majority of countries around the world in recent decades (Florio et al., 2020; Van Dyke et al., 1999-2013; Saha et al., 2016). Even though extensive surgery is by far the most effective therapy, the 5-year rate of survival with CCA ranged between 20% and 40% following resection (van der Gaag et al., 2012; Nagino et al., 2013; Mazzaferro et al., 2020). Due to the lack of clinical symptoms, about 70% of patients have missed out on surgical resection at diagnosis time (Banales et al., 2020). Thus, it is necessary to find sensitive and specific biomarkers for CCA.

**FIGURE 1:**
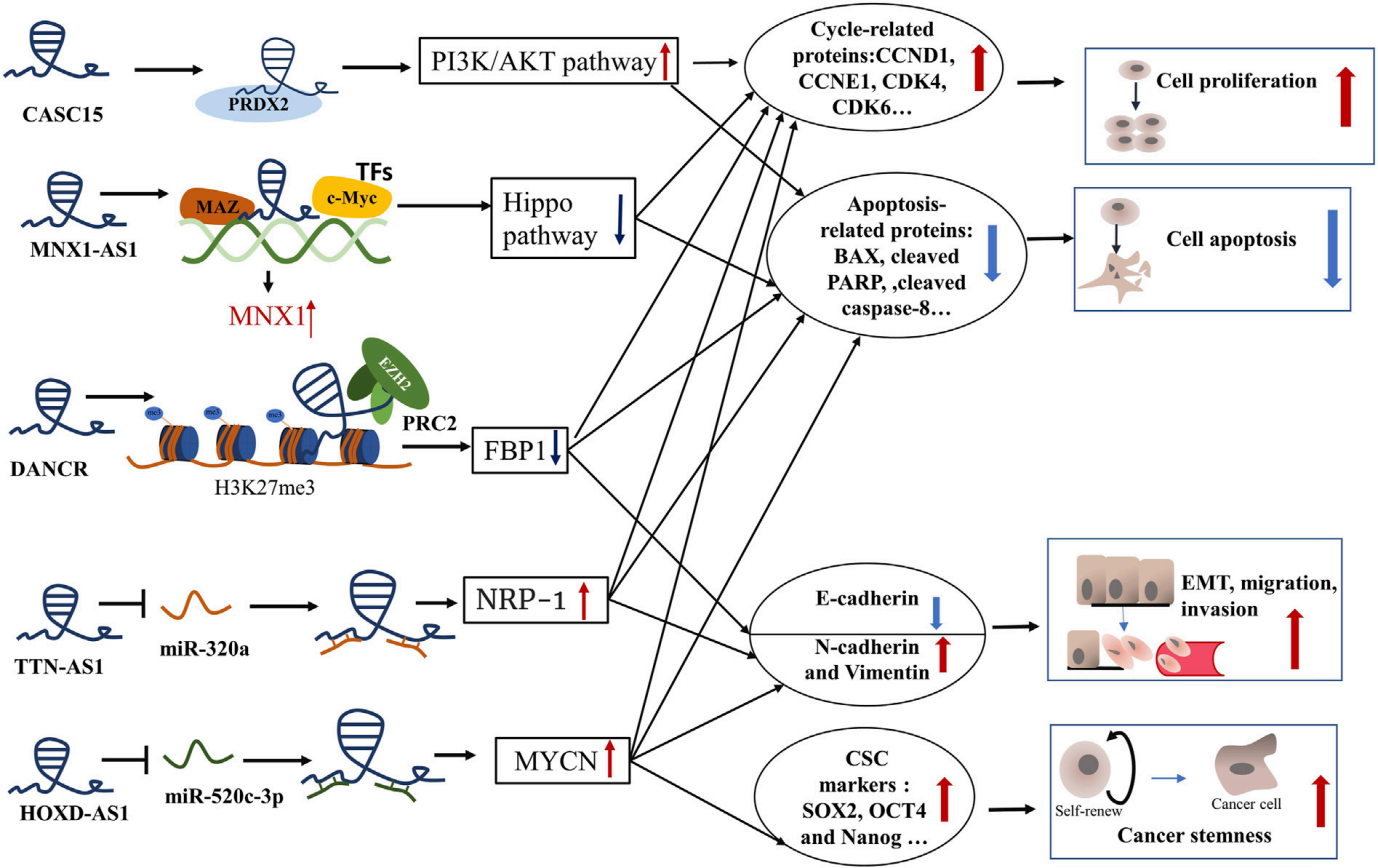
The role and mechanism of lncRNAs in CCA progression. CASC15, cancer-associated susceptibility 15 gene; PRDX2, peroxiredoxin 2; CCND1, cyclin D1; CCNE1, cyclin E1; CDK4, cyclin dependent kinase 4; CDK6, cyclin dependent kinase 6; MNX, motor neuron and pancreas homeobox protein 1; MNX1-AS1, MNX1 antisense RNA 1; MAZ, Myc-associated zinc finger protein; TFs, transcription factors; BAX, BCL2 associated X;PARP, poly ADP-ribose polymerase; DANCR, differentiation antagonizing nonprotein coding RNA; EZH2, enhancer of zeste homolog 2; H3K27me3, methylation of lysine 27 on histone 3; PRC2, polycomb repressive complex 2; FBP1, Fructose-1, 6-biphosphatase; TTN-AS1, titin-antisense RNA1; NRP-1, neuropilin-1; HOXD-AS1, HOXD cluster antisense RNA 1; MYCN, MYCN proto-oncogene, bHLH transcription factor; SOX2, SRY-box transcription factor 2; OCT4, organic cation/carnitine transporter4.

Long non-coding RNA (lncRNA) constitutes a non-coding RNA longer than 200 nucleotides and was previously thought to be translational noise since it lacks the ability to code for proteins (Kopp and Mendell, 2018). Nevertheless, mounting evidence indicates that lncRNAs are involved in carcinogenesis or tumor suppression (Rinn and Chang, 2020). LncRNAs are obtained by assembling factors and Pol II at promoters, followed by intron elongation and splicing, and finally by adding a methylated guanosine cap and polyadenylated tail (Guo et al., 2016). LncRNAs have extensive functions in different biological processes, involving chromatin modification, gene transcription, and translation (Quinn and Chang, 2016). LncRNAs serve extensive functions according to their cellular location. In the nucleus, the functions of lncRNAs mainly include chromatin interaction, RNA processing, and transcriptional regulation, whereas lncRNAs in the cytoplasm can interact with miRNAs, functioning as competitive endogenous RNAs (ceRNAs) to modulate mRNA and cellular signaling pathways (Schmitt and Chang, 2016). The dysregulation of lncRNAs has been widely observed to be remarkably linked to the development of cancers, consisting of liver cancer, gastrointestinal cancer, lung cancer, breast cancer, and hematological malignancies (Lv et al., 2017; Kim et al., 2018; Wu et al., 2019; Zhuo et al., 2019; Wang et al., 2020a; Pan et al., 2020). A lot of abnormally expressed lncRNAs have been discovered between CCA and adjacent normal tissues (Long et al., 2019). The purpose of this review is to summarize the role and underlying mechanism of lncRNAs in CCA progression (Figure 1; Table 1) and explore their potential as biomarkers and therapeutic targets.

**TABLE 1:** Some oncogenic lncRNAs in cholangiocarcinoma.

LncRNA	Location	Expression	Target	Signaling pathway	Function	References
CASC15	6p22.3	Up	PRDX2	CASC15/PRDX2/PI3K/AKT	Proliferation↑, migration↑, invasion↑	Zhang et al. (2021)
DANCR	4q12	Up	miR-345-5p EZH2	DANCR/miR-345-5p/Twist1	Proliferation↑, migration↑, invasion↑, EMT↑	Wang et al. (2019); Zhu et al. (2020b)
H19	11p15.5	Up	miR-612	HIF1α/H19/miR-612/Bcl-2	Proliferation↑, migration↑, invasion↑, EMT↑	Xu et al. (2017a); Yu et al. (2020a)
HOTAIR	12q13.13	Up	miR-204-5p	HOTAIR/miR-204-5p/HMGB1	Proliferation↑, migration↑, invasion↑, EMT↑	Qin et al. (2018); Lu et al. (2020)
HOXD-AS1	2q31.1	Up	miR-520c-3p	SP1/HOXD-AS1/miR-520c-3p/MYCN	Proliferation↑, migration↑, invasion↑, EMT↑, stemness↑	Li et al. (2020b)
LINC01410	9q13	Up	miR-124-3p	LINC01410/miR-124-3p/smad5	Proliferation↑, migration↑, Invasion↑	Jiang et al. (2020b)
LMCD1-AS1	3p26.1	Up	miR-345-5p	E2F1/LMCD1-AS1/miR-345-5p/COL6A3	Proliferation↑, invasion↑	Yu et al. (2019)
MALAT1	11q13.1	Up	miR-204	MALAT1/miR-204/CXCR4; MLAT1/PI3K/Akt	Proliferation↑, migration↑, invasion↑, EMT↑	Tan et al. (2017); Wang et al. (2017)
MNX1-AS1	7q36.3	Up	c-Myc MAZ	MNX1-AS1/c-Myc and MAZ/MNX1/Ajuba/Hippo	Proliferation↑, migration↑, invasion↑	Li et al. (2020a)
NEAT1	11q13.1	Up	miR-186-5p EZH2	NEAT1/miR-186-5p/PTP4A1/PI3K/AKT	Proliferation↑, migration↑, invasion↑, EMT↑	Zhang et al. (2018c); Li et al. (2021b)
NNT-AS1	5p12	Up	miR-485 miR-203	NNT-AS1/miR-485/BCL9; NNT-AS1/miR-203/PI3K/AKT and ERK1/2	Proliferation↑, migration↑, invasion↑, EMT↑	Huang et al. (2019a); Gu et al. (2019); Gu et al. (2020)
PAICC	3q22.2	Up	miR-141-3p miR27a-3p	PAICC/miR-141-3p/YAP1; PAICC/miR27a-3p/YAP1	Proliferation↑, migration ↑, invasion↑	Xia et al. (2020)
PVT1	8q24.21	Up	miR186 EZH2	PVT1/miR186/KLF5; SOX2/PVT1/miR-186/SEMA4D	Proliferation↑, migration↑, invasion↑	Yu et al. (2018); Yu et al. (2020b)
SPRY4-IT1	5q31.3	Up	KLF2 LATS2 miR-101-3p	SP1/SPRY4-IT1/KLF2 and LATS2; SP1/SPRY4-IT1/miR-101-3p/KLF2 and LATS2	Proliferation↑, migration↑, invasion↑ EMT↑	Xu et al. (2018b)
TTN-AS1	2q31.2	Up	miR-320a	TTN-AS1/miR-320a/neuropilin-1	Proliferation↑, migration↑, invasion↑, EMT↑	Zhu et al. (2020a)
UCA1	19p13.12	Up	miR-122 AKT	UCA1/miR-122/CLIC1/ERK/MAPK; UCA1/AKT/GSK-3β/CCND1	Proliferation↑, migration↑, invasion↑, EMT↑	Xu et al. (2017d); Kong et al. (2019)
ZEB1-AS1	10p11.22	Up	miR-133b miR-200a	AR/ZEB1-AS1/miR-133b/HOXB8; ZEB1-AS1/miR-200a/ZEB1	Proliferation↑, migration↑, invasion↑, EMT↑	Jiang et al. (2020a); Jiao et al. (2020)

EMT, epithelial-mesenchymal transition; CASC15, cancer-associated susceptibility 15 gene; PRDX2, peroxiredoxin 2; PI3K/AKT, phosphoinositide 3-kinase/AKT; DANCR, differentiation antagonizing nonprotein coding RNA; EZH2 enhancer of zeste homolog 2; HOXD-AS1, HOXD cluster antisense RNA 1; SP1, specificity protein 1; MYCN, MYCN proto-oncogene bHLH transcription factor; LMCD1-AS1, LMCD1 antisense RNA 1; COL6A3, collagenVI-alpha3 chain; MALAT1, metastasis-associated lung adenocarcinoma transcript 1; CXCR4, chemokine receptor-4; MNX1, motor neuron and pancreas homeobox protein 1; MNX1-AS1, MNX1 antisense RNA 1; MAZ, Myc-associated zinc finger protein; NEAT1, nuclear paraspeckle assembly transcript 1; PTP4A1, protein Tyrosine Phosphatase 4A1; NNT-AS1, nicotinamide nucleotide transhydrogenase-antisense RNA1; BCL9, B-cell CLL/lymphoma 9 protein; PAICC, prognostic-associated ICC; YAP1, yes-associated protein; PVT1, Plasmacytoma variant translocation 1; KLF5, kruppel like factor 5; SOX2, SRY-box transcription factor 2; SEMA4D, semaphorin 4D; SPRY4-IT1, SPRY4 intronic transcript 1; KLF2, kruppel like factor 2; LATS2, large tumor suppressor kinase 2; TTN-AS1, TTN antisense RNA 1;UCA1, urothelial cancer associated 1; CLIC1, chloride intracellular channel 1; MAPK, Mitogen-activated protein kinase; AKT, AKT serine/threonine kinase 1; GSK-3β, glycogen synthase kinase 3 beta; CCND1, cyclin D1; ZEB1, zinc finger E-box binding homeobox 1; ZEB1-AS1, ZEB1 antisense RNA 1; AR, androgen receptor; HOXB8, homeobox B8.

## The Role of lncRNAs in Cholangiocarcinoma Progression

### Proliferation and Anti-Apoptosis

The infinite proliferation of tumor cells is due to abnormal modulation of the cell cycle and anti-apoptosis. Knockdown of these upregulated oncogenic lncRNAs in CCA can induce cell cycle arrest and apoptosis by regulating cycle-related proteins and apoptosis-related proteins. Some key lncRNA-related signaling pathways were also involved in the pathogenesis of cholangiocarcinoma. For example, silencing lncRNA CASC15 hampered the expression of cell cycle regulators (CCND1, CCNE1, CDK4, and CDK6) and enhanced apoptosis-related proteins (BAX, cleaved PARP, and cleaved caspase-8) to suppress ICC cell cycle progression and promote cell apoptosis (Zhang et al., 2021). Further, the mechanism experiments showed that lncRNA CASC15 modulated the cell cycle by binding to the protein peroxiredoxin 2 (PRDX2) to active the phosphoinositide 3-kinase (PI3K)/AKT signaling pathway (Zhang et al., 2021). In addition, lncRNAs can also recruit transcription factors to promote the tumorigenesis and progress of CCA. The Hippo pathway has a significant impact on mammalian organ size and participates in the development of various cancers (Ma et al., 2019). Li et al. (2020a) reported that lncRNA MNX1-AS1 bound to transcription factors (TFs) c-myc and MAZ to induce the expression of MNX1 in ICC cells. Then MNX1 prohibited the Hippo pathway by promoting the expression of Ajuba protein, which contributed to the cell proliferation of ICC (Li et al., 2020a). Besides, one of the most fundamental mechanisms of lncRNA tumorigenesis is chromatin remodeling in CCA. Research reported that lncRNA DANCR cooperated with the enhancer of zeste homolog 2 (EZH2) to inhibit the tumor suppressor gene FBP1 expression epigenetically through methylation of lysine 27 on histone 3 (H3K27me3), contributing to CCA growth *in vitro* and *in vivo* (Wang et al., 2019). Moreover, lncRNA can act as a ceRNA for miRNA and subsequently modulate proliferation-related and apoptosis-related mRNA in CCA, informing the lncRNA-miRNA-mRNA regulation network. The NF-κB pathway exerts an oncogenic role by boosting the expression of oncogenes involving considerable antiapoptotic genes (Sun, 2017). By inhibition of miR-140, lncRNA SNGH1 facilitated the expression of Toll-like receptor 4(TLR4) mRNA and protein and activate the NF-κB pathway to motivate CCA tumor growth (Li et al., 2019a). Collectively, lncRNAs can regulate CCA cell proliferation and apoptosis through binding to protein, recruiting TFs, chromatin remodeling, and sponging miRNA.

### Migration and Invasion

Epithelial-mesenchymal transition (EMT) is a procedure in which tightly attached polarized epithelial cells obtain the phenotype of mesenchymal cells, allowing them to spread and colonize new territory (Gugnoni and Ciarrocchi, 2019). Therefore, EMT is a vital mechanism for tumor metastasis and invasion. Several lncRNAs, such as CCAT1, CCAT2, HOTAIR, TTN-AS1, ASAP1-IT1, DANCR, LINC00261, LINC00665, and SPRY4-IT1, have a close connection with EMT. These lncRNAs enhanced the migration along with the infiltration of CCA by increasing vimentin and N-cadherin expression but reducing E-cadherin expression (Zhang et al., 2017a; Xu et al., 2018a; Xu et al., 2018b; Guo et al., 2018; Qin et al., 2018; Zhu et al., 2020a; Zhu et al., 2020b; Gao et al., 2020; Lu et al., 2021). Most lncRNAs affect CCA cell migration, invasion, and EMT *via* the mechanism of ceRNA to target miRNAs, such as CCAT1, CCAT2, HOTAIR, TTN-AS1, DANCR, and SPRY4-IT1. The transforming growth factor β (TGF-β) pathway is critical for EMT (David et al., 2016). Zhu et al. (2020a) first reported that lncRNA TTN-AS1 upregulated neuropilin-1 (NRP-1) through sponging miR-320a. Further mechanistic studies manifested that neuropilin-1 as an important co-receptor activated the HGF/c-Met and TGF-β/TGF-βRI pathways contributing to progression, EMT, and angiogenesis in CCA cells (Zhu et al., 2020a). Guo et al. (2018) observed that the silence of lncRNA ASAP1-IT1 has the significant action of inhibiting CCA cell migration, proliferation, and reversing EMT through blocking the hedgehog signaling pathway. And rescue experiments demonstrated that the repressed EMT progress was motivated again by upregulating hedgehog-related Smo and Gli1 (Guo et al., 2018). However, the underlying mechanism that ASAP1-IT1 activates the hedgehog pathway remains unclear. CLIC1 can act as an essential motivator in cancer migration and invasion. It is reported that lncRNA-UCA1 enhanced the ability of CCA cell migration and invasion through inducing CLIC1 targeted by miR-12 and positively regulating the ERK/MAPK signaling pathway (Kong et al., 2019). On the other hand, some lncRNAs, such as CASC2, lnc-LFAR1, MIR22HG, and MEG3, act as protectors to restrain CCA growth and metastasis (Li et al., 2019b; Chen et al., 2019; Hu et al., 2019; Peng et al., 2020). For instance, highly expressed lncRNA CASC2 stimulated the expression of E-cadherin while decreasing the expression of N-cadherin and Vimentin by sponging miR-18a to induce SOCS5, thereby reversing the EMT process (Peng et al., 2020).

### Cancer Stemness

Cancer stem cells (CSCs) have the capacity to self-renew and develop into a variety of different types of cancer cells, playing a key role in tumor chemosensitivity and recurrence. A small number of highly expressed lncRNAs, including lnc-PKD2-2-3, ZEB1-AS1, HOXD-AS1, and LINC00665, have participated in the regulation of CCA stemness (Qiu et al., 2019; Jiang et al., 2020a; Li et al., 2020b; Lu et al., 2021). Many studies report that cancer cell stemness has a strong effect on chemoresistance in various cancers (Smith and Macleod, 2019). For instance, the overexpression of lnc-PKD2-2-3 enhanced CSC markers (CD44, CD133, and OCT4) expression and sphere generation efficiency in CCA cell lines (Qiu et al., 2019). Moreover, lnc-PKD2-2-3 was observed to have been dramatically upregulated in CCA stem-like cells, which illustrated that lnc-PKD2-2-3 might act as a fresh CSC marker (Qiu et al., 2019). ZEB1-AS1, HOXD-AS1and LINC00665 enhanced the cancer cell stemness respectively through sponging miR-133b, miR-520c-3p, and miR-424-5p (Jiang et al., 2020a; Li et al., 2020b; Lu et al., 2021). So far, these lncRNAs function as ceRNA for miRNAs, which is the major mechanism is ceRNA to modulate tumor cell stemness in CCA cells. However, the mechanism and singling pathways of lncRNA on CCA cell stemness are complex and unclear, which need to be explored in depth.

### Angiogenesis and Oxidative Stress

Recently, more attention has been paid to the vital function of the tumor microenvironment in tumor progression, which includes angiogenesis, oxidative stress, and immune escape (Whiteside, 2008). Vascular endothelial growth factor (VEGF) is considered to be an essential factor in regulating tumor angiogenesis (Siveen et al., 2017). The content of lncRNA DANCR was enriched in CCA cells and tissues. Downregulation of DANCR inhibited the levels of VEGF-C and VEGF-A in cholangiocarcinoma cell lines (Zhu et al., 2020b). It is also reported that lncRNA SNHG6 reinforced CCA proliferation and angiogenesis by modulating miR-101-3p-targeted E2F8 which is indispensable for tumor angiogenesis (Wang et al., 2020b). There is a common opinion that chronic inflammation and bile duct epithelial cell injury can facilitate CCA tumorigenesis and growth (Sirica et al., 2021). Oxidative stress-induced lncRNA HULC, along with H19, increased the level of inflammatory mediators IL-6 and CXCR4, which in turn activated the positive feedback pathway of inflammation, promoting tumorigenesis in CCA *via* working as a sponge of miR-372/miR-373 and let-7a/let-7b, respectively (Wang et al., 2016). In terms of the tumor microenvironment, research on the role and mechanism of lncRNA is still limited in CCA.

## LncRNAs as Diagnostic and Prognostic Biomarkers in Cholangiocarcinoma

The value of tumor biomarkers lies in their ability to provide early diagnosis, predict disease prognosis, monitor disease recurrence, and offer guidance for disease treatment. Unfortunately, there is no effective and reliable biomarker for the diagnosis, as well as prognosis of cholangiocarcinoma. The most commonly used biomarkers are carbohydrate antigen 199 (CA199) and carcinoembryonic antigen (CEA) in the clinical diagnosis of CCA, whether used alone or in combination, but their value is limited due to the inconsistency in sensitivity (47.2%–98.2%) and specificity (89.7%–100%) (Tshering et al., 2018; Macias et al., 2019). Interestingly, many lncRNAs localized in exosomes can be secreted into bodily fluids (for instance, plasma, urine, and bile) as circulating RNA with high tissue specificity, becoming putative non-invasive biomarkers for many tumors (Di Meo et al., 2017; Xu et al., 2018c; Lapitz et al., 2020). However, there is little research about the diagnostic value of lncRNAs from exosomes or EVs in CCA. Ge et al. (2017) observed that exosomal lncRNAs of ENST0000588480.1 and ENST00000517758.1, which were isolated and identified from human bile samples, in contrast with normal individuals, showed significantly higher expression in CCA patients. The diagnostic value of these lncRNAs was examined in CCA, and the result showed that the combination of ENST00000588480.1 and ENST00000517758.1 harbored more excellent sensitivity versus CA199 (82.9% vs. 74.3%) (Ge et al., 2017). Notably, the level of both lncRNAs was negatively associated with survival time (Ge et al., 2017). In a recent study, a few lncRNAs were identified in serum and urine extracellular vesicles (EVs) from patients with CCA, primary sclerosing cholangitis (PSC), and healthy individuals to evaluate their prognostic value (Lapitz et al., 2020). In serum EVs, MALAT-1 and LOC100190986 possessed a high accuracy for the differentiation of CCA and PSC with the area under the curve (AUC) values of both 1.0 (Lapitz et al., 2020). In urine EVs, LOC100134868 displayed an excellent diagnostic value with an AUC of 0.896 (patients with CCA vs. healthy individuals) (Lapitz et al., 2020). Moreover, the diagnostic ability of some other lncRNAs in plasma or tissue, including PCAT1, CPS1-IT1, H19, PVT1, and NEF (Han et al., 2018; Shi et al., 2018; Liang et al., 2019), has also been verified in CCA.

In the last decade, it was found that many dysregulated lncRNAs in CCA tissues and cells were linked to the pathological characteristics and prognosis of patients with cholangiocarcinoma (Merdrignac et al., 2021). Xie et al. (2021) established a prediction model with five lncRNAs (HULC, AL359715.5, AC006504.8, AC090114.2, and AP00943.4) to distinguish the CCA patients with poor prognosis. In the validation cohort, the five-lncRNA module had a perfect predictive value with a AUC of 0.816 for prognosis in CCA. However, the cohort studies on the predictive effect of lncRNAs are still lacking. Most of the lncRNA-related research has focused on the mechanism and function of dysregulated lncRNAs in the tissues and cells of CCA. The following lncRNAs have been documented to be linked to the prognosis of cholangiocarcinoma (Table 2): ANRIL (Angenard et al., 2019), ASAP1-IT1 (Guo et al., 2018), CCAT1 (Zhang et al., 2017a), CCAT2 (Xu et al., 2018a), CRNDE (Xia et al., 2018), FOXD2-AS1 (Hu et al., 2022), GAPLINC (Wang et al., 2018), H19 (Xu et al., 2017a), HOTAIR (Qin et al., 2018), HOTTIP (Gao et al., 2021), HOXD-AS1 (Li et al., 2020b), LINC00261 (Gao et al., 2020), LINC00665 (Lu et al., 2021), LINC00667 (Li et al., 2021a), LINC01296 (Zhang et al., 2018a), LOXL1-AS1 (Zhang et al., 2019a), NNT-AS1 (Huang et al., 2019a), MALAT1 (Tan et al., 2017), PAICC (Xia et al., 2020), PANDAR (Xu et al., 2017b), PCAT1 (Sun et al., 2021), lnc-PKD2-2-3 (Qiu et al., 2019), SNHG3 (Tian et al., 2019), Sox2ot (Li et al., 2018), TUG1 (Xu et al., 2017c), UCA1 (Xu et al., 2017d), ZEB1-AS1 (Jiang et al., 2020a), ZFAS1 (Li et al., 2019c).

**TABLE 2:** Prognostic-related lncRNAs in cholangiocarcinoma.

LncRNA	Sample	Expression	Clinicopathological characteristic	OS	AUC	References
ANRIL	Tissue	Up	Tumor size	√	-	Angenard et al. (2019)
ASAP1-IT1	Tissue	Up	Lymph node invasion, TNM stage, postoperative recurrence	√	-	Guo et al. (2018)
CCAT1	Tissue	Up	Lymph node invasion, TNM stage	√	0.831	Jiang et al. (2017)
CCAT2	Tissue	Up	Lymph node invasion, TNM stage, microvascular invasion	√	0.702	Bai et al. (2018)
CRNDE	Tissue	Up	Poor differentiation, tumor size, lymph node invasion, TNM stage	√	-	Xia et al. (2018)
FOXD2-AS1	Tissue	UP	Lymph node invasion, TNM stage	√	0.7406	Hu et al. (2021)
GAPLINC	Tissue	Up	Lymph node invasion, TNM stage	√	0.713	Wang et al. (2018)
H19	Tissue	Up	Tumor size, TNM stage, postoperative recurrence	√	-	Xu et al. (2017a)
HOTAIR	Tissue	Up	Lymph node invasion, TNM stage, postoperative recurrence	√	-	Qin et al. (2018)
HOTTIP	Tissue	Up	Lymph node invasion, distant metastasis	√	-	Gao et al. (2021)
HOXD-AS1	Tissue	Up	Lymph node invasion, TNM stage	√	0.786	Li et al. (2020b)
LINC00261	Tissue	Up	Tumor size, lymph node invasion, TNM stage, postoperative recurrence	√	-	Gao et al. (2020)
LINC00665	Tissue	Up	Lymph node invasion, distant metastasis, TNM stage	√	-	Lu et al. (2021)
LINC00667	Tissue	Up	Lymph node invasion, TNM stage	√	0.830	Li et al. (2021a)
LINC01296	Tissue	Up	Lymph node invasion, TNM stage	√	-	Zhang et al. (2018a)
LOXL1-AS1	Tissue	Up	Lymph node invasion, TNM stage	√	-	Zhang et al. (2019a)
MALAT1	Tissue, plasma	Up	Tumor size, pathological T stage, perineural invasion	√	-	Tan et al. (2017); Shi et al. (2018)
NNT-AS1	Tissue	Up	Tumor size, lymph node invasion, TNM stage	√	-	Huang et al. (2019a)
PAICC	Tissue	Up	Tumor number, tumor size, vascular invasion	√	-	Xia et al. (2020)
PANDAR	Tissue	Up	Lymph node invasion, TNM stage, postoperative recurrence	√	-	Xu et al. (2017b)
PCAT1	Tissue	Up	Lymph node invasion, TNM stage	√	0.823	Sun et al. (2021)
PKD2-2-3	Tissue	Up	Poor differentiation, TNM stage	√	-	Qiu et al. (2019)
SNHG3	Tissue	Up	Lymph node invasion, distant metastasis, TNM stage	√	-	Tian et al. (2019)
Sox2ot	Tissue	Up	Lymph node invasion, TNM stage, postoperative recurrence	√	-	Li et al. (2018)
TUG1	Tissue	Up	Tumor stage, intrahepatic metastasis, lymph node metastasis, perineural invasion	√	-	Xu et al. (2017c)
UCA1	Tissue	Up	Lymph node invasion, TNM stage, postoperative recurrence	√	-	Xu et al. (2017d)
ZEB1-AS1	Tissue	Up	Lymph node invasion, TNM stage	√	0.749	Jiang et al. (2020a); Jiao et al. (2020)
ZFAS1	Tissue	Up	Lymph node invasion, TNM stage, postoperative recurrence	√	-	Li et al. (2019c)

Clinicopathological characteristic: clinicopathological characteristics significantly correlated to the corresponding lncRNA expression were filled in the table; OS (overall survival): the level of the corresponding lncRNA expression that was strongly associated with overall survival of CCA patients was noted as “√”; AUC (area under the curve): the AUC of the corresponding lncRNA was used to evaluate its prognostic efficiency by ROC curve and the uncertain AUC was noted as “-”.

OS, overall survival; AUC, area under the curve; TNM, tumor node metastasis; ANRIL, CDKN2B antisense RNA 1; ASAP1-IT1, ASAP1 intronic transcript 1; CCAT1, colon cancer associated transcript 1; CCAT2, colon cancer associated transcript 2; CRNDE, colorectal neoplasia differentially expressed; FOXD2-AS1, FOXD2 adjacent opposite strand RNA 1; GAPLINC, gastric adenocarcinoma predictive long intergenic non-coding RNA; HOTAIR, HOX transcript antisense RNA; HOTTIP, HOXA transcript at the distal tip; HOXD-AS1, HOXD cluster antisense RNA 1; LOXL1-AS1, LOXL1 antisense RNA 1; NNT-AS1, NNT antisense RNA 1; MALAT1, Metastasis-associated lung adenocarcinoma transcript 1; PAICC, prognostic-associated ICC; PANDAR, promoter of CDKN1A antisense DNA damage activated RNA; PCAT1, prostate cancer associated transcript 1; SNHG3, small nucleolar RNA host gene 3; SOX2-OT, SOX2 overlapping transcript; TUG1, taurine upregulated 1; UCA1, urothelial cancer associated 1; ZEB1-AS1, ZEB1 antisense RNA 1; ZFAS1, ZNFX1 antisense RNA 1.

In this review, these lncRNAs for diagnosis and/or prognosis, including PCAT1, MALAT1, H19, NEF, CCAT1, CCAT2, ZEB1-AS1, will be described in detail.

### Prostate Cancer-Associated Transcript 1

Prostate cancer-associated transcript 1(PCAT1) was initially identified as an overexpressed lncRNA in prostate cancer tissues (Prensner et al., 2011), which has also been proved to be abnormally expressed in various malignant tumors and to play a carcinogenic role (Wen et al., 2016; Bi et al., 2017; Xu et al., 2017e; Huang et al., 2018; Liu et al., 2019a; Shen et al., 2019). Additionally, dysfunctional lncRNA PCAT1, identified in body fluids, can be used as a prospective biomarker for esophageal squamous cell carcinoma, bladder carcinoma, and multiple myeloma (Shen et al., 2017; Huang et al., 2019b; Zhang et al., 2019b). Zhang et al. (2017b) discovered that PCAT1 was remarkably elevated in extrahepatic cholangiocarcinoma (ECC) tissues and cell lines, and could regulate ECC progression through the Wnt/β-catenin signaling pathway. An updated study uncovered that YY1-induced lncRNA PCAT1 promoted the progression of cholangiocarcinoma through the miR-216a-3p/BCL3 axis (Sun et al., 2021). Also, this study showed that the overexpressed PCAT1 was confirmed as the factor associated with the adverse outcome for CCA patients and made an effective prognostic prediction with an AUC of 0.823 (Sun et al., 2021). In terms of its diagnostic value, a study illustrated that the AUC of plasma PCAT1 was 0.784 by ROC (receiver operating characteristic curves), which indicated that circulating PCAT1 may act as an early diagnostic biomarker for HCC (Shi et al., 2018). In general, PCAT1 has the potential value in the diagnosis and prognosis of CCA, but clinical trials on a large scale are still needed to confirm it.

### Metastasis-Associated Lung Adenocarcinoma Transcript 1

Metastasis-associated lung adenocarcinoma transcript 1 (MALAT1) is a highly conserved lncRNA with a length of about 8.5 kb transcribed from human chromosome 11q13 in the nucleus (Ji et al., 2003; Eißmann et al., 2012). MALAT1, which was detected in body fluids, has been identified as a valuable biomarker either alone or in combination with other molecules in malignant tumors (Goyal et al., 2021), such as nasopharyngeal carcinoma (He et al., 2017), osteosarcoma (Huo et al., 2017), and bladder cancer (Zhan et al., 2018). In CCA, MALAT1 was established to be upregulated in tissues and cells, and over-expression of MALAT1 was remarkably correlated with adverse clinical features (Tan et al., 2017). Shi et al. (2018) showed that MALAT1 in plasma has excellent specificity and sensitivity as a diagnostic biomarker of hilar cholangiocarcinoma (AUC, 0.860; sensitivity, 81.1%; specificity, 90.9%). Further, when PCAT1, MALAT1, along with CPS1-IT1 were combined, the sensitivity and specificity increased to 85.5% and 93.2%, respectively (Shi et al., 2018).

### H19

LncRNA H19 is a long-chain noncoding RNA transcribed by a paternally imprinted carcinoembryonic gene on chromosome 11p15.5. The content of H19 was elevated in fetal tissues and downregulated in various healthy tissues after birth (Gabory et al., 2010). A pooled analysis indicated that H19 may be a reliable biomarker to discriminate patients with tumors from normal individuals (Liu et al., 2019b). In cholangiocarcinoma, aberrant expression of H19 has been shown to promote proliferation and invasion through inflammatory and oxidative stress pathways *in vitro* (Wang et al., 2016; Xu et al., 2017a; Yu et al., 2020a). Besides, overexpression of H19 was a dismal predictor for CCA patients, which was significantly correlated with tumor size, TNM stage, as well as postoperative recurrence (Xu et al., 2017a). Remarkably, H19 was observed to have moderate sensitivity in distinguishing CCA tissues from normal ones with an AUC of 0.7422 (Han et al., 2018). Furthermore, a combination of AC005550.3, H19, C3P1, PVT1, along with LPAL2 showed more excellent sensitivity (93.75%) and specificity (81.25%) with an AUC of 0.8828 in differentiating CCA tissues from normal ones (Han et al., 2018). Thus, the establishment of an effective lncRNA-related identification panel plays an essential role in improving the detection rate of CCA.

### Neighboring Enhancer of FOXA2

LncRNA neighboring enhancer of FOXA2 (NEF) was first discovered as a tumor suppressor in hepatocellular carcinoma by reversing the EMT process and inhibiting tumor metastasis (Liang et al., 2018). In intrahepatic cholangiocarcinoma, a study reported that lncRNA-NEF was dramatically downregulated in tumor tissues and upregulated lncRNA-NEF repressed cell migration and invasion by inhibition of runt-related transcription factor 1 (RUNX1) (Liang et al., 2019). Additionally, the study observed that the level of plasma lncRNA-NEF in patients (including patients at stage I or II) was significantly lower than in healthy controls (Liang et al., 2019). Plasma lncRNA-NEF exhibited excellent ability for early diagnosis with an AUC of 0.8642 (Liang et al., 2019). Therefore, plasma lncRNA-NEF may become the effective early diagnostic biomarker of intrahepatic cholangiocarcinoma.

### Colon Cancer-Associated Transcript 1 and Colon Cancer-Associated Transcript 2

Colon cancer-associated transcript 1 (CCAT1) was initially identified as being overexpressed in colon cancer tissues, peripheral blood, and related lymph node tissues, but it was almost undetectable in normal human body tissues (Nissan et al., 2012). As a rising star among carcinogenic lncRNAs, CCAT1 has been closely related to many cancers’ occurrence, development, and drug resistance (Jin et al., 2020; Shan et al., 2020; Su et al., 2020; Wang et al., 2021). CCAT1 expression was dramatically increased in CCA tissues and was shown to be strongly linked to lymph node infiltration and late TNM stage (Zhang et al., 2017a; Jiang et al., 2017). Through multivariate analysis, it was established that increased CCAT1 expression is an independent prognostic factor and that it has a certain sensitivity (81.8%) and specificity (74.5%) for estimating overall survival *via* ROC analysis (Jiang et al., 2017). Xu et al. (2018a) and Bai et al. (2018) both documented that the level of CCAT2 expression was inversely linked with the overall survival rate of CCA patients. The study suggested that CCAT2 has practicalvaluein predicting the prognosis of patients with ICC, with an AUC of 0.702 and 0.715 for OS and PFS, respectively (Bai et al., 2018). Besides, a meta-analysis in 2019 showed that upregulated CCAT lncRNA families, especially CCAT2, can predict shorter overall survival (*p* < 0.001) (Dai et al., 2019). Therefore, CCAT1 and CCAT2 can be considered promising prognostic biomarkers of CCA.

### ZEB1 Antisense RNA 1

ZEB1-AS1 is an antisense transcript originating from the promoter of Zinc finger E-box binding homeobox 1(ZEB1), a prominent factor in the EMT process of tumor epithelial cells (Li et al., 2016; Caramel et al., 2018). ZEB1-AS1 plays a carcinogenic factor in various digestive system tumors, for instance, hepatocellular carcinoma, esophageal cancer, colorectal cancer, and gastric cancer (Li et al., 2016; Zhang et al., 2018b; Zhao et al., 2019; Wu et al., 2020). Jiang et al. (2020a) proved that ZEB1-AS1 was overexpressed in CCA and promoted CCA growth along with metastasis *in vivo*, as well as *in vitro* experiments. Patients with high ZEB1-AS1 expression were clearly related to lymph node invasion, progressed TNM stage, along with the shorter survival time when compared to the low ZEB1-AS1 expression group (Jiang et al., 2020a). Regarding prognostic efficiency, the AUC of ZEB1-AS1 was 0.749 with 65.5% sensitivity and 80.0% specificity by ROC analysis (Jiang et al., 2020a). Hopefully, ZEB1-AS1 could become a sensitive, as well as specific prognostic biomarker for CCA.

## The Role of lncRNAs in the Therapeutic Target of Cholangiocarcinoma

**FIGURE 2:**
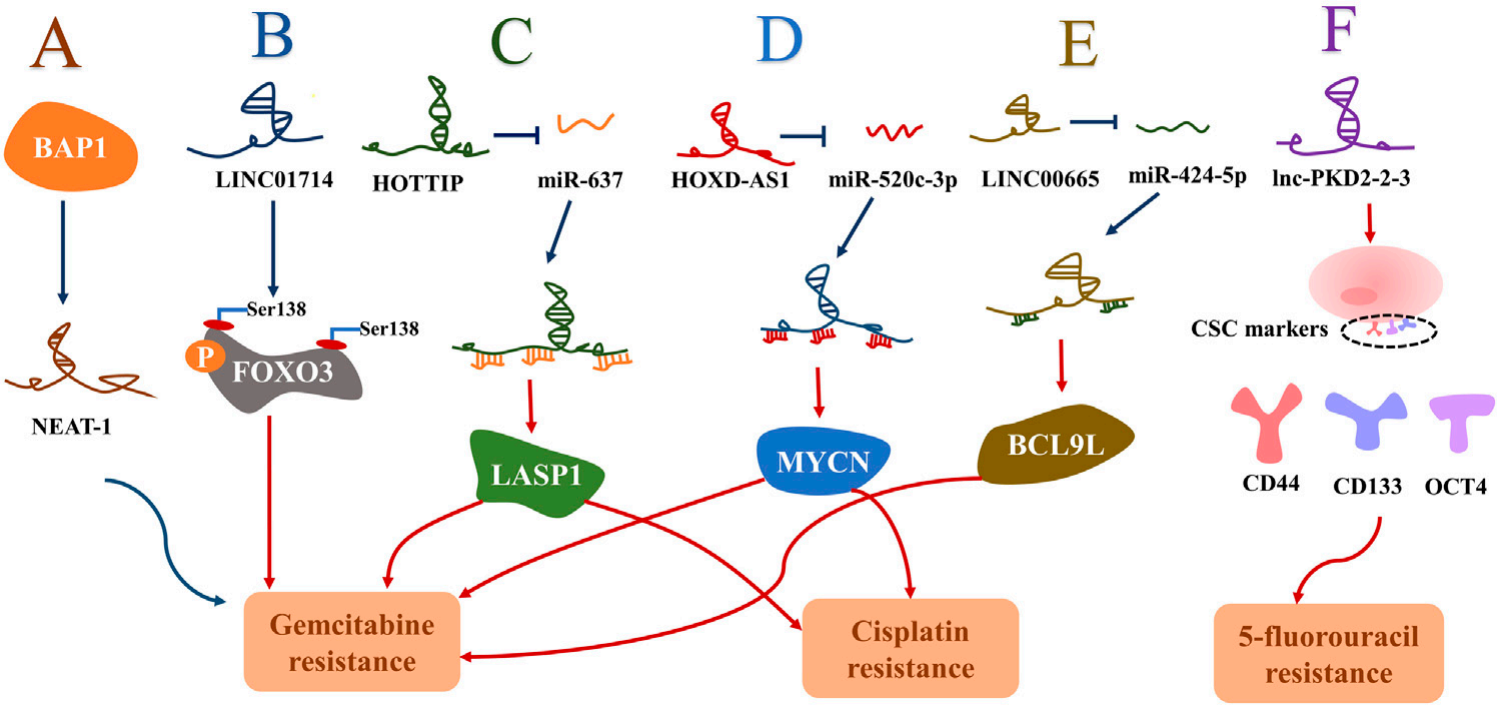
The chemo-resistance mechanism of lncRNAs in cholangiocarcinoma. Note: the blue arrow represents a negative correlation and the red arrow represents a positive correlation. **(A)**: NAET-1 negatively regulated by BAP1 promoted the sensitivity of CCA cells to gemcitabine. **(B)**: LINC01714 facilitated gemcitabine sensitivity by reducing phosphorylated FOXO3-Ser318. **(C,D)**: HOTTIP and HOXD-AS1 promoted the gemcitabine and cisplatin resistance through sponging miR-637 to increase LASP1 and through sponging miR-520c-3p to increase MYCN, respectively. **(E)**: LINC00665 enhanced BCL9L to promote gemcitabine resistance by sponging miR-424-5p. **(F)**: lnc-PKD2-2-3 enhanced 5-FU resistance by facilitating cell stemness in CCA. Abbreviation: BAP1, BRCA-1 associated protein-1; NEAT-1, nuclear paraspeckle assembly transcript 1; FOXO3, Forkhead Box O3; HOTTIP, HOXA transcript at the distal tip; MYCN, MYCN proto-oncogene bHLH transcription factor; HOXD-AS1, HOXD cluster antisense RNA 1; LASP1, LIM and SH3 domain protein 1; BCL9L, B cell CLL/lymphoma 9-like; CSC, cancer stem cell; CD44, Cluster of differentiation-44; CD133, Cluster of differentiation-133; OCT4, octamer-binding transcription factor 4.

**FIGURE 3:**
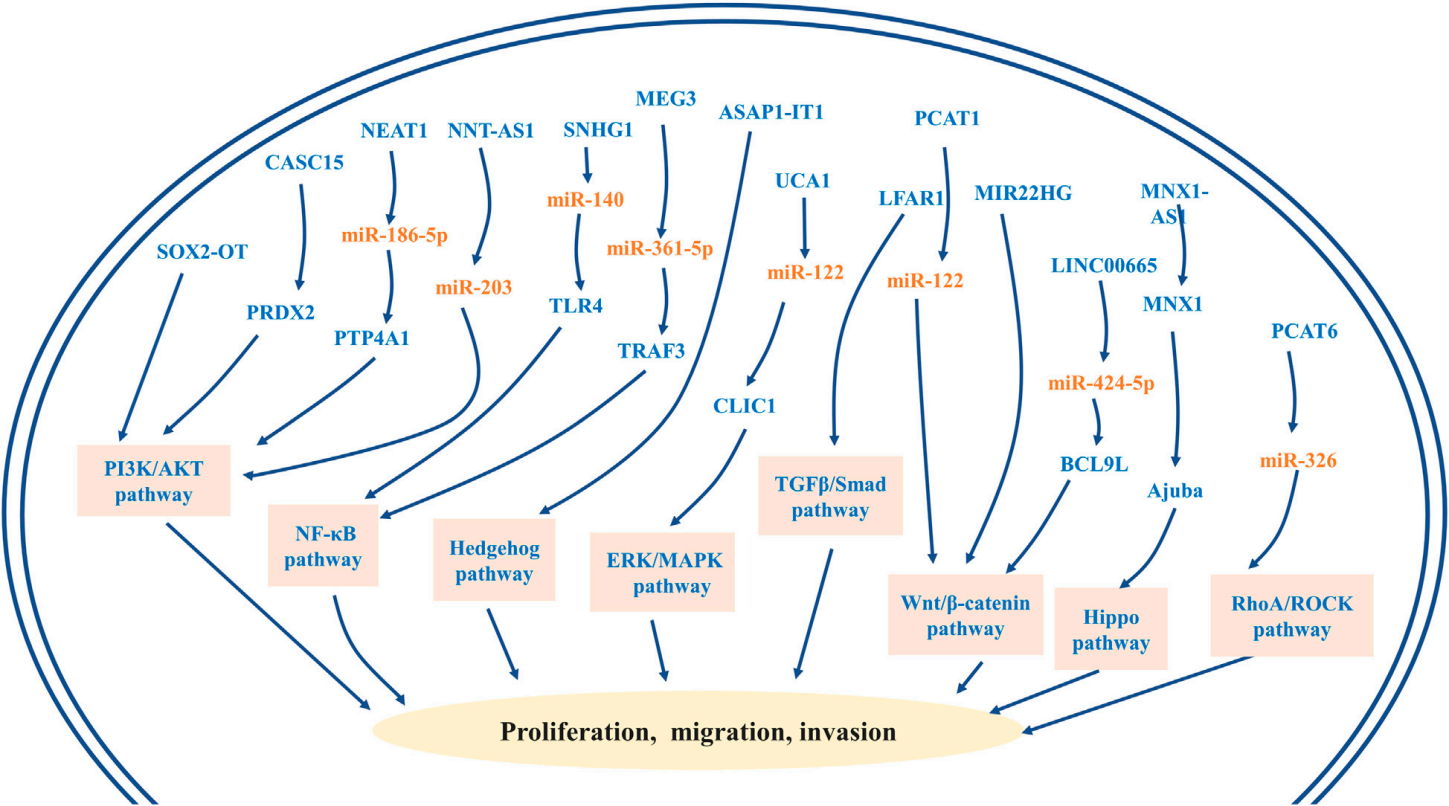
LncRNA-related oncogenic signaling pathways and their roles in the progression of cholangiocarcinoma, including PI3K/AKT, NF-κB, Hedgehog, Wnt/β-catenin, ERK/MAPK, TGFβ/Smad, Hippo, and RhoA/ROCK pathways.

R0 radical surgical resection has remained the mainstay of potentially curative treatment (Valle et al., 2021). For individuals with advanced or unresectable cancer, systemic chemotherapy is the primary treatment option (Rizvi et al., 2018). Gemcitabine plus cisplatin constitutes the standard first-line chemotherapy for advanced cholangiocarcinoma (Valle et al., 2010). However, the median overall survival for this standard chemotherapy regimen is less than 1 year (Weigt and Malfertheiner, 2010). Recently, studies have identified lncRNAs as important players in CCA chemoresistance. So far, a few novel lncRNAs, including NEAT-1, LINC01714, HOXD-AS1, HOTTIP, LINC00665, and lnc-PKD2-2-3, have been revealed to modulate chemoresistance in CCA (Figure 2) (Lu et al., 2021; Qiu et al., 2019; Li et al., 2020b; Gao et al., 2021; Parasramka et al., 2017; Shen et al., 2020). These resistance-associated lncRNAs present new opportunities for the treatment of CCA. It was reported that both NEAT-1 and LINC01714 could positively regulate the sensitivity to gemcitabine in CCA cells. More specifically, Parasramka et al. (2017) noticed that gemcitabine cytotoxicity was strengthened in low BAP1 expressing CCA cells, and then NEAT1 was enriched in CCA cells after the knockout of BAP1. Further experiments revealed that BAP1-targeted lncRNA NEAT1 could enhance the responsivity of CCA cells to gemcitabine (Figure 2A) (Parasramka et al., 2017). Additionally, overexpressed LINC01714 facilitated the susceptibility of CCA cells to gemcitabine by reducing phosphorylated FOXO3-Ser318 (Figure 1B), which indicated that LINC01714 transcription promoter and gemcitabine combined with chemotherapy may have a synergistic effect on the treatment of advanced CCA patients (Shen et al., 2020). LncRNA HOTTIP and HOXD-AS1 were reported to regulate the sensitivity of CCA cells to gemcitabine and cisplatin through sponging miR-637 to increase LASP1 and sponging miR-520c-3p to increase MYCN, respectively (Figures 2C,D) (Li et al., 2020b; Gao et al., 2021). The acquisition of EMT and the existence of CSC are not only crucial mechanisms in the invasion and metastasis of CCA, but recent investigations have established that they may be closely linked to the chemoresistance of CCA (Huang et al., 2019c; Varamo et al., 2019). Lu et al. (2021) proved that LINC00665 can act as a ceRNA to modulate the miR-424-5p/BCL9L axis, thereby promoting EMT and cell stemness and finally enhancing gemcitabine resistance of CCA (Figure 2E). It is the first report that lnc-PKD2-2-3 enhanced drug resistance to 5-FU in CCA cells possibly by promoting CCA stemness (Figure 2F) (Qiu et al., 2019). Therefore, the above-mentioned upregulated lncRNAs may serve as biomarkers for chemotherapeutic drug resistance to guide clinical chemotherapy medication, and may also provide new therapeutic targets for cholangiocarcinoma. Moreover, Tu et al. (2020) first observed that the overexpression of PCAT6 can promote macrophage M2 polarization to repress the immune response of macrophages *via* sponging miR-326 and activating RhoA/ROCK pathways. *In vitro*, it was found that the tumor growth of PCAT6 knockout mice was significantly inhibited. Therefore, targeting PCAT6 may become potentially feasible immunotherapy for CCA treatment (Tu et al., 2020). Some lncRNAs may become underlying therapeutic targets by modulating previously mentioned signal pathways, including the PI3K/AKT, NF-κB, Hedgehog, Wnt/β-catenin, ERK/MAPK, TGFβ/Smad, Hippo, and RhoA/ROCK pathway (Figure 3). Although no drug has been developed to target lncRNA directly, several approaches have been successfully used to deplete specific oncogenic lncRNAs in cells, including RNA interference (RNAi) and antisense oligonucleotides (ASOs) (Lennox and Behlke, 2016). Of note, the prospect of lncRNA-targeted therapy is bright thanks to a deep dive into the mechanisms of lncRNAs and the development of emerging technologies.

## Conclusion and Future Perspectives

Cholangiocarcinoma is a kind of malignant tumor with an adverse prognosis and growing prevalence. With the advancements in high-throughput sequencing and molecular gene technologies, it has been demonstrated that aberrantly expressed lncRNAs harbor diverse functions in the progression of malignancies, including CCA. However, the underlying molecular mechanism of most lncRNAs remains unclear in CCA. Thus, more basic biological research will be required. A rising number of improperly expressed lncRNAs have been detected in tissue, and serum and bile secretion of CCA patients. Due to their extensive distribution and relatively stable structure, lncRNAs harbor the potential to be extremely useful biomarkers for monitoring, diagnosis, along with prognosis. At present, aberrant expression of many lncRNAs in CCA tissues has been observed to be remarkably linked to adverse clinical features and OS, which also means that lncRNAs may be used as effective indicators for risk-stratification and predicting survival in CCA patients. Furthermore, the combination of several exosomal lncRNAs in bile and serum has shown excellent sensitivity and specificity for the diagnosis in CCA. Therefore, the exosomal lncRNA will become a rising star in CCA diagnosis and prognosis, and more studies should focus on exploring specific exosomal lncRNAs as reliable biomarkers. However, due to a lack of significant sample clinical cohort verification, the value of lncRNAs as clinical biomarkers is controversial in CCA. In addition, several lncRNAs, as biomarkers of chemotherapy resistance, can be used to guide clinical chemotherapy. Many lncRNAs are the critical molecules involved in activating multiple carcinogenic signaling pathways in CCA. Therefore, lncRNA is the novel promising therapeutic target for CCA.
