# Corrigendum: Long non-coding RNAs as molecular biomarkers in cholangiocarcinoma

**DOI:** 10.3389/fcell.2022.1090025

**Published:** 2022-11-17

**Authors:** Yanhua Wu, Khizar Hayat, Yufei Hu, Jianfeng Yang

**Affiliations:** ^1^ Department of Gastroenterology, The Fourth School of Clinical Medicine, Zhejiang Chinese Medical University, Hangzhou, China; ^2^ Department of Gastroenterology, International Education College of Zhejiang Chinese Medical University, Hangzhou, China; ^3^ Department of Gastroenterology, Affiliated Hangzhou First People’s Hospital, Zhejiang University School of Medicine, Hangzhou, China; ^4^ Key Laboratory of Integrated Traditional Chinese and Western Medicine for Biliary and Pancreatic Diseases of Zhejiang Province, Hangzhou, China

**Keywords:** cholangiocarcinoma, lncRNA, biomarker, diagnosis, prognosis, therapeutic target

In the published article, there was an error in **Affiliation 1**. Instead of “The Fourth Clinical College of Zhejiang Chinese Medicine University,” it should be “The Fourth School of Clinical Medicine, Zhejiang Chinese Medical University.”

The authors apologize for this error and state that this does not change the scientific conclusions of the article in any way. The original article has been updated.

